# A hybrid modeling approach for assessing mechanistic models of small molecule partitioning in vivo using a machine learning-integrated modeling platform

**DOI:** 10.1038/s41598-021-90637-1

**Published:** 2021-05-27

**Authors:** Victor Antontsev, Aditya Jagarapu, Yogesh Bundey, Hypatia Hou, Maksim Khotimchenko, Jason Walsh, Jyotika Varshney

**Affiliations:** VeriSIM Life, 1 Sansome Street, Suite 3500, San Francisco, CA 94104 USA

**Keywords:** Biophysics, Computational biology and bioinformatics, Drug discovery, Systems biology

## Abstract

Prediction of the first-in-human dosing regimens is a critical step in drug development and requires accurate quantitation of drug distribution. Traditional in vivo studies used to characterize clinical candidate’s volume of distribution are error-prone, time- and cost-intensive and lack reproducibility in clinical settings. The paper demonstrates how a computational platform integrating machine learning optimization with mechanistic modeling can be used to simulate compound plasma concentration profile and predict tissue-plasma partition coefficients with high accuracy by varying the lipophilicity descriptor logP. The approach applied to chemically diverse small molecules resulted in comparable geometric mean fold-errors of 1.50 and 1.63 in pharmacokinetic outputs for direct tissue:plasma partition and hybrid logP optimization, with the latter enabling prediction of tissue permeation that can be used to guide toxicity and efficacy dosing in human subjects. The optimization simulations required to achieve these results were parallelized on the AWS cloud and generated outputs in under 5 h. Accuracy, speed, and scalability of the framework indicate that it can be used to assess the relevance of other mechanistic relationships implicated in pharmacokinetic-pharmacodynamic phenomena with a lower risk of overfitting datasets and generate large database of physiologically-relevant drug disposition for further integration with machine learning models.

## Introduction

Pharmacokinetic (PK) predictions of compound disposition are critical for safety and efficacy assessments both before and after drugs enter clinical trials. These predictions are predicated on a thorough understanding of compound absorption, distribution, metabolism, and excretion (ADME). Compounds are required to be characterized thoroughly for ADME properties prior to regulatory approval, and during preclinical drug discovery/development act as key attributes that can determine the compound’s prioritization for further testing^1^. The standard in vivo assay for ADME characterization is the measurement of plasma concentrations over time after an administered dose of a molecule in subjects^2^. This gives insight into critical parameters driving compound pharmacokinetics; specifically, the standard metrics for PK outputs are area under the curve (AUC), maximum concentration (C_max_), time of maximum concentration (t_max_), area under the moment curve (AUMC), steady-state volume of distribution (Vd_ss_), mean residence time (MRT), and half-life (t_1/2_). These output metrics are quantified using non-compartmental modeling and considered comprehensive for capturing compound PK behavior in vivo^3^.

### Current paradigm in drug development testing

Existing methodologies for PK characterization typically rely on in vivo studies in mice, rats and dogs^4^. Animal models are the current gold standard for conducting these PK predictions, however, they suffer from an overall low relevance to in vivo clinical studies, as evidenced by the current 92 percent failure rates of compounds that enter the clinic; and of those approximately 16 percent of compound failures are attributed to ADME-related issues in clinical trials^5^. These studies are expensive and time-consuming and are known to suffer from translatability issues^6^. Even so, the standard paradigm for preclinical ADME data translation includes: (1) testing a compound in preclinical subjects and collect outputs, (2) building 1- or 2-compartment model of compound PK, or use physiologically-based pharmacokinetic models (PBPK), (3) fitting model parameters to the compound PK data, (4) scaling optimized parameters to human-relevant values using established allometry functions or according to surface area/body weight, and (5) simulating outcomes from an established dosing regimen^7^.

These developed models are typically tested only once human studies commence. The learnings from the resulting outcome are frequently discarded or not utilized to help inform further model development. The resulting challenge is that these models, although fit to experimental datasets, are rarely improved upon and developed with the clinical outcome in mind^3, 6, 8, 9^.

An additional challenge with the standard paradigm of studying only systemic PK outputs is the loss of insight into tissue-specific kinetics that can drive compound safety/tox dynamics^10^. Apart from some indications and mechanisms of action realized in the blood vessel bed, the site of action for the majority of compounds is a non-circulatory tissue or organ system necessitating more specific prediction of drug permeation to a site-of-action implicated in target binding^11^. Therefore, determination of the tissue:plasma partition coefficients (Kp) for organs and tissues is critical because these properties of drug compounds defines their exposure to the specific receptors. Due to differences in Kp values for different organs, drug target exposure may not be directly correlated with general plasma concentration metrics including Vd_ss_. While VD_ss_ shows distribution of the drug compounds between plasma and tissues, it does not count differences of tissue composition and morphology. However, these experiments are expensive and time-consuming^12^. As such, several in silico methods have been developed to predict Kp values from more easily obtained in vitro data. Using a combination of tissue composition information and the compound’s physicochemical characteristics, such as lipophilicity (logP) and the unbound fraction in plasma (Kp), these methods account for the distribution of the drug between water and drug-binding components, including proteins, lipids, and phospholipids. That is amplified as predictions of drug disposition and effects are becoming more personalized to subjects with conditions directly influencing clinical PK of pharmaceuticals^13^. In vivo patient stratification can present practical and ethical challenges, and thus benefits from accurate translation of insights generated via preclinical studies^14^. When resolving the challenge of poor preclinical translatability, a crucial aspect is understanding which mechanistic behaviors are modulating the compound PK. If datasets consist only of the calculated endpoints (AUC, C_max_, t_max_, Vd_ss_), it is both challenging to personalize prediction for a specific patient or to leverage advances in statistical learning such as machine learning and deep learning (ML/DL) models for the structure-based prediction of PK parameters^15, 16^.

### Computational predictions of drug distribution

For drugs with particularly narrow therapeutic window, it is particularly important to determine precisely which route of administration (ROA) is optimal as well as maximum tolerated dose (MTD) and minimum effective concentration (MEC) for the specific route. Optimizing an ROA and compound formulation is predicated on, for a given dose, minimizing compound distribution to organs where a toxic burden is suspected and maximizing penetration to a site-of-action. These characteristics fall into the domain of distribution. Overall, tracking the disposition of different active components of therapies (parent and metabolites) not only systemically but to general active and inactive sites is critical for continued model improvement in PK.

There has been a multitude of mathematic relationships developed over the last few decades that explore mechanistic model-based predictions of compound distribution into tissues with Kp values^17^. There are different model modalities, such as compartment-based models that characterize organs as well-perfused and well-mixed, finite element analysis (FEA), and three-dimensional recapitulations of tissue structures^18, 19^. In PK contexts mechanistic models of distribution into tissue components (neutral lipids, phospholipids, intracellular water, etc.) are most frequently used to output predictions of tissue:plasma partition coefficients. The more well-characterized approaches are described in works by Poulin and Thiel, Berezhkovskiy, Rodgers and Rowland (Rodgers), and Schmitt et al.^20–23^. Each of these equations focuses on key assumptions of how compound physicochemical properties and specific physiological parameters interact to present themselves in this organ-specific equilibrium constant. Poulin and Theil model proposes a tissue:plasma partition coefficient prediction that accounts for dissolution into water and nonspecific binding to neutral lipids and phospholipids. Berezhkovskiy’s modified method assumes that only drugs in the water fraction bind to tissues. Rodgers and Rowland approach considers above mentioned approached but also counts the impact of drug ionization on partitioning^24^. In cases where mechanistic equations are not used to calculate compartment-specific organ partition coefficients, global optimizations of Vd_ss_ are performed. As mentioned before, this is a useful indicator for relative compound distribution but lacks the insight to develop next-generation dose optimizations.

### Identifiability in computational models

**Figure 1:**
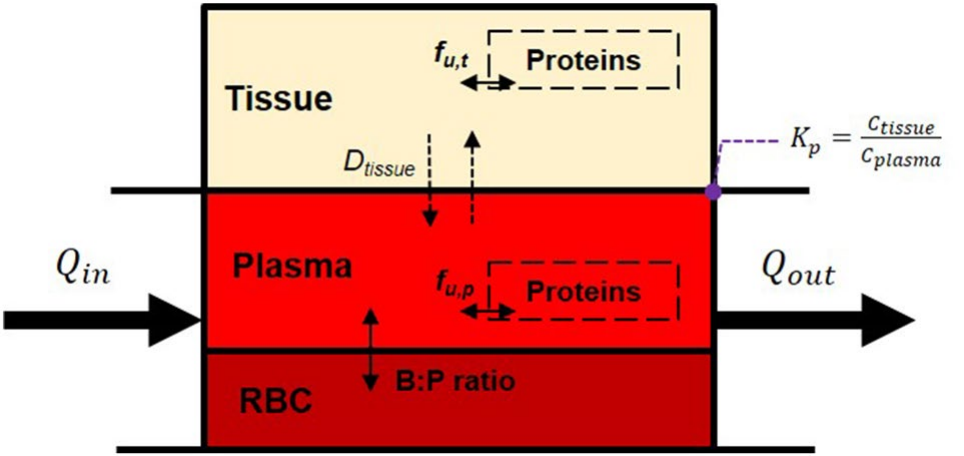
Basic diagram of quasi-2D tissue distribution in a compartmental model. D_tissue_ is the diffusion coefficient for a compound across the plasma-tissue barrier; fu refers to unbound drug concentration in plasma (p) and tissue (t), and Q refers to blood flow rates through a tissue section.

Information from any mathematical model is obtained primarily through parameter inference and predictions on the trajectories of the internal states of the system from experimentally determined measurements. However, a major challenge in extracting this information (especially in the case of biological systems) deals with handling measurements that are not feasible due to experimental limitations. This theoretical phenomenon, i.e. the ability to infer the parameters of the biological system from the observed outputs/measurements is called identifiability^25, 26^. One such use case relates to predicting the organ-specific drug distribution in PBPK models^12^. Understanding the site/organ-specific drug distribution is important for evaluating the drug efficacy, safety and insights into other biological mechanisms that are dependent on the drug concentration in that tissue/organ. Since most available in vivo PK data relate to plasma concentrations, obtaining the organ-specific drug distribution remains a significant problem due to identifiability issues as discussed above. The problem is augmented especially in cases where the site or target of action for the drug is in organs or compartments other than plasma. One approach to overcome this problem is by employing mechanistic frameworks either through incorporating mechanisms that govern the physical/chemical interactions between the drug and the tissue or through empirical equations that predict drug distributions based on observations from other cases. Building physiological mechanisms is entirely dependent on the problem under study, i.e. subject to certain drugs and organs of interest and the particular physics underpinning the mechanism of distribution. On the other hand, predictions from empirical equations can be used for any drug or organ of interest based on the behavior of other drugs. Key examples of empirical equations that can predict organ-specific drug distribution are the Rodgers-Rowland (Rodgers) and Poulin equations^21, 22^. The predictions are not solely based on empirical relationships as certain physical parameters such as the lipophilicity (logP), degree of ionization/nature of the compound (p*K*a) and protein binding in plasma and tissues (fup) are employed in evaluating the degree of drug distribution. Even though these empirical equations provide us a decent initial estimate on drug distribution values, we cannot rely on them exclusively for accurate predictions. This is because we do not have a complete understanding of how parameters like p*K*a and logP present themselves in a physiological context for driving the drug distribution between plasma and the site of action. The datasets that were used to validate these relationships tend to be decoupled from systemic studies that are more numerous in availability. Different tissues have different levels of lipids/phospholipids that are highly dependent on the subject or population under consideration and we do not have a good estimate or bounds for these values to predict the drug distribution. Also, certain parameters such as the lipophilicity (logP) for a compound is usually evaluated through octanol:water systems which might not an efficient way to translate to in vivo systems as biological lipids have different partitioning coefficients. Some of the relevant mechanisms and approximations driving distribution are captured in Fig. 1; specifically, these implicate binding of compounds to tissue proteins and Fickian (passive) diffusion of free compound into tissue depots as a result of concentration gradients. Boundary conditions are assumed to be driven by equilibrium partition coefficients across a barrier. All of these factors play a role in distribution, and, effectively, both volume of distribution and organ-specific drug disposition. Therefore, using direct optimization of the Kp values for various organ compartments may be considered more beneficial in comparison to mechanistic models and LogP optimization since it does not count the limitations imposed by suggested lipophilicity, degree of ionization, and protein binding. Therefore, the goal of the present study was to estimate accuracy of the PK simulation using AI-based prediction of the Kp values in comparison to generally accepted mechanistic models.

In this work, we used an AI/ML-based PK-PD modeling platform BIOiSIM and its functionalities to resolving identifiability challenges present in PK distribution simulations through hybrid integration of existing mechanistic models and physicochemical parameter optimization. The selected approach is evaluated for a proof-of-principle application of the BIOiSIM platform to the simulation of drug disposition in vivo for one small molecule compounds, and comparing simulation outputs using a fixed optimization for distribution based on the Rodgers equation to a global optimization of an effective tissue:plasma partition coefficient^27, 28^. The accuracy of plasmavenous concentration simulation is evaluated across both methods, and insights as well as limitations of the study are discussed.

## Results

### Sensitivity and convergence testing

**Figure 2:**
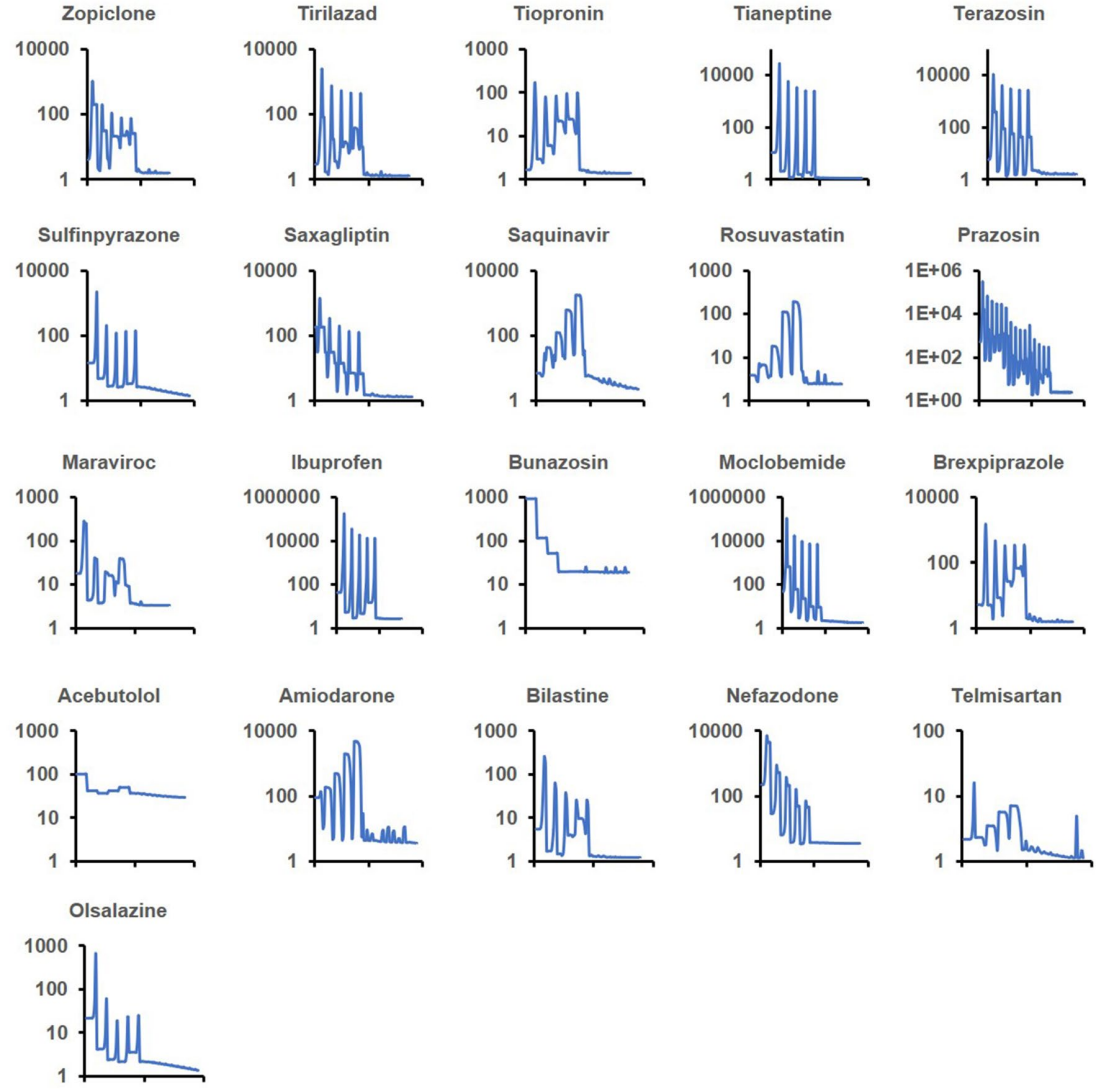
logP optimization convergence plots for 21 small molecule compounds tested with logP optimization. Y-axis corresponds to the cost function; X-axis is the cost progression across grid search optimization (high oscillation) and descent (minimal oscillation) simulation trials.

The core BIOiSIM model was used in conjunction with existing in vivo datasets to optimize distribution parameters and missing PK parameters—specifically, blood:plasma ratio and first-order absorption rate constant, *k*_*a*_ (h^−1^). The high amplitude oscillations in convergence plots (Fig. 2) correspond to the large steps taken during coarse optimization. Post-selection of the optimal coarse parameter combinations, each of the simulations converged as evidenced by the flat tail of each optimization curve. Overall, there is high confidence in the optimized parameter values as a result of the minimal variation of objective function value at the end of optimization. The final optimized datapoints are expressed in Table S1 for the different configurations that were tested.

### Simulation accuracy

**Figure 3:**
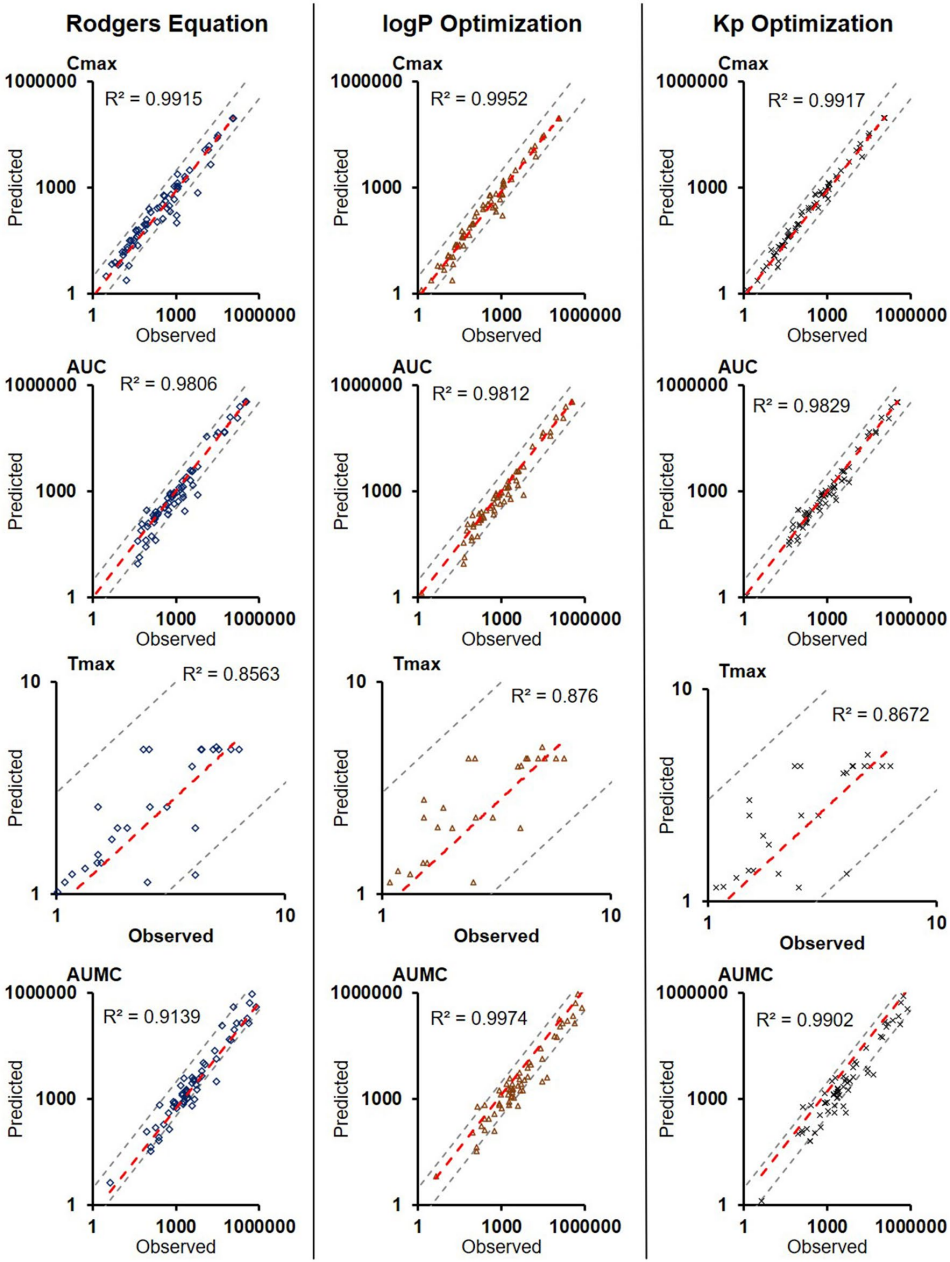
Plots of Observed vs. Predicted PK metrics across the different optimization conditions for firstt-order PK outputs. Red lines correspond to lines of best fit, gray lines are bounds of ± threefold-error.

**Figure 4:**
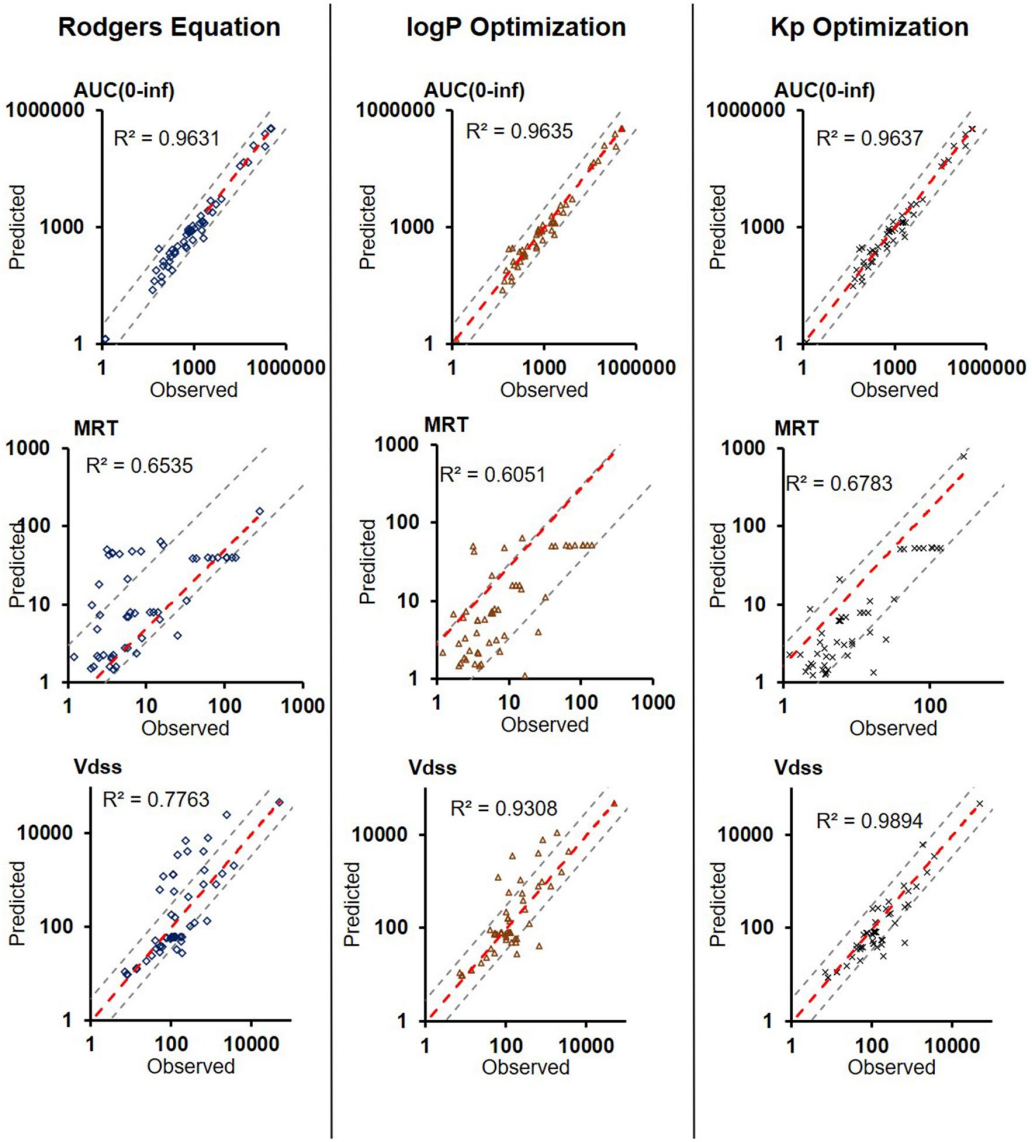
Plots of Observed vs. Predicted PK metrics across the different optimization conditions for second-order PK outputs. Red lines correspond to lines of best fit, gray lines are bounds of ± threefold-error.

**Figure 5:**
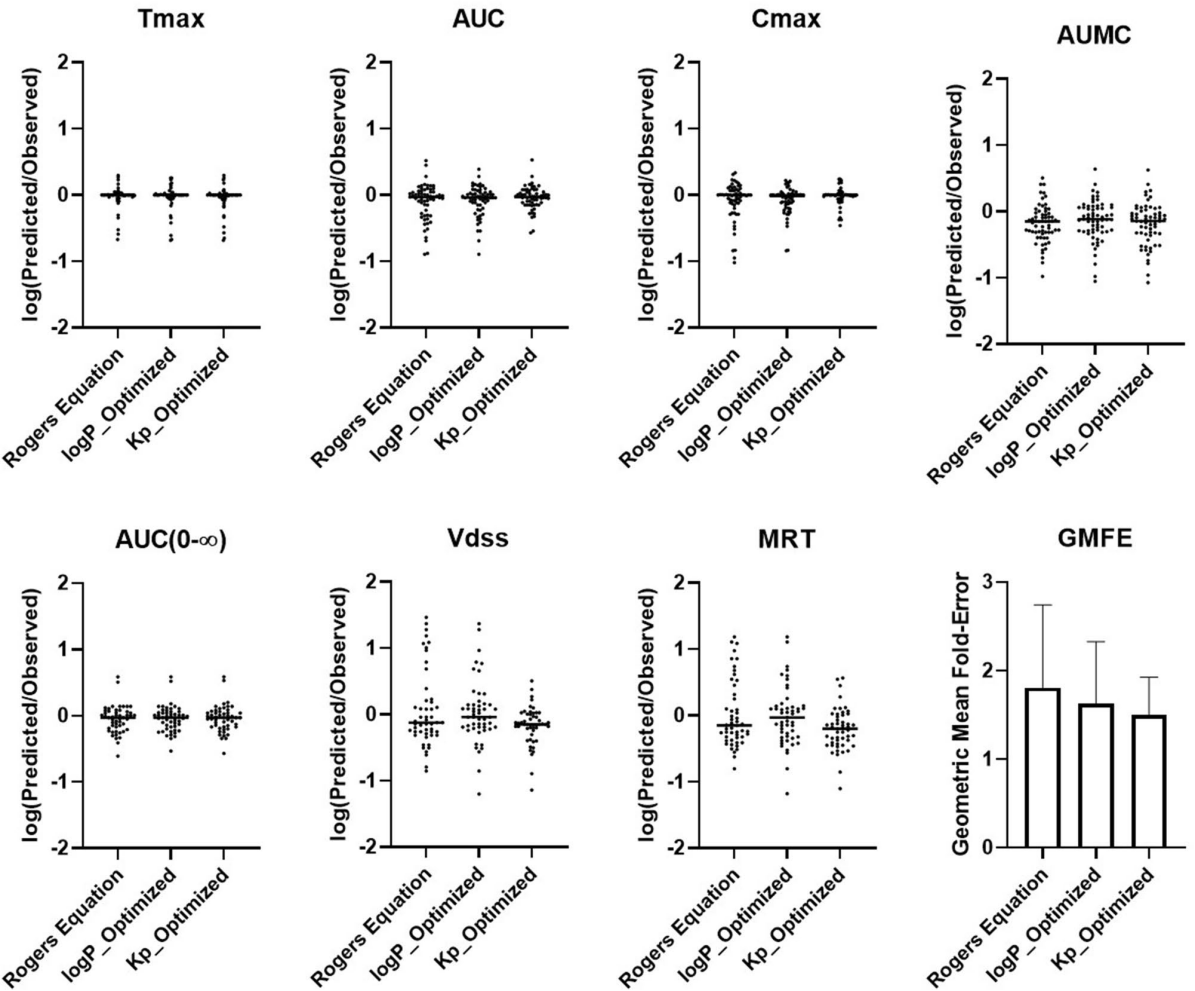
Comparison of the PK outputs fold-difference magnitude and geometric mean fold-error (GMFE) across the three optimization conditions.

The comparison of BIOiSIM simulation accuracy is captured in Figs. 3, 4, 5 and Table 1. The key PK outputs discussed previously were assessed for accuracy using AFE, AAFE and r^2^ as well as an overall Geometric Mean Fold Error prediction of accuracy for each compound (Fig. 5). The Pearson correlation coefficient values are consistently high across all metrics and optimization conditions (0.6051–0.9974), indicative of good agreement between observed PK outputs and the simulated ones. For first-order PK outputs (AUC_0–t_, C_max_, T_max_) AAFE < 1.6, indicating that the plasma concentration simulations are of comparable magnitude to those from experimental studies. Additionally, the second-order outputs (AUMC, AUC_0–inf_, MRT, Vd_ss_) have comparably low AAFE values for Kp and logP-optimized outputs (1.74, 1.36, 2.11, 2.13 for logP; 1.85, 1.37, 2.02, 1.71 for Kp optimization). This is indicative of the model having sufficient mechanistic complexity for capturing the PK disposition of the different molecules.

**Table 1:** Assessment of predicted PK parameter values in subjects across different methodologies for establishing partition coefficients. Compounds without sufficient terminal-phase data were excluded from AUC_0–∞_, MRT, and Vd_ss_ analysis.

Metric	Distribution Configuration	AFE	AAFE	% < 3-Fold AAFE	r^2^
C_max_, μg/L N = 63	Rogers Equation	0.82	1.51	90%	0.9915
	logP_Optimized	0.84	1.34	97%	0.9952
	Kp_Optimized	0.96	1.20	100%	0.9917
T_max_, h N = 63	Rogers Equation	0.92	1.21	94%	0.8563
	logP_Optimized	0.91	1.24	95%	0.876
	Kp_Optimized	0.92	1.22	95%	0.8672
AUC_0–t_, μg*h/L N = 63	Rogers Equation	0.78	1.52	90%	0.9806
	logP_Optimized	0.82	1.42	94%	0.9812
	Kp_Optimized	0.89	1.30	97%	0.9829
AUMC, μg*h*h/L N = 63	Rogers Equation	0.65	1.84	84%	0.9139
	logP_Optimized	0.71	1.74	89%	0.9974
	Kp_Optimized	0.63	1.85	79%	0.9902
MRT, h N = 52	Rogers Equation	1.04	2.54	68%	0.654
	logP_Optimized	0.95	2.11	80%	0.6051
	Kp_Optimized	0.61	2.02	82%	0.6785
Vd_ss_, L N = 52	Rogers Equation	1.20	2.63	70%	0.7766
	logP_Optimized	1.07	2.13	76%	0.9315
	Kp_Optimized	0.69	1.71	84%	0.9905

**Figure 6:**
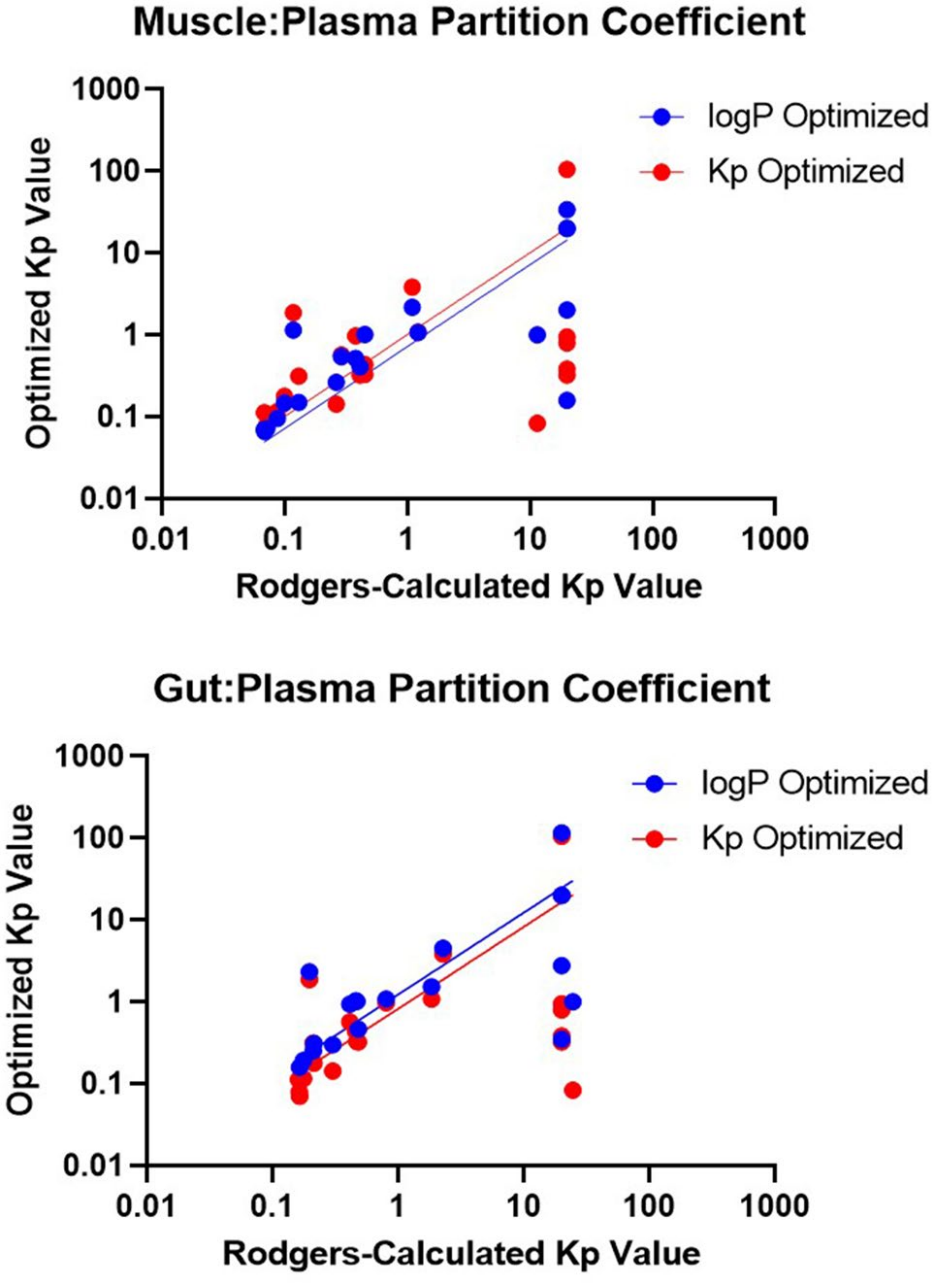
Comparison of the simulated Kp values to the Rodgers-calculated values for gut and muscle compartments with linear regression best-fit.

Between the optimization conditions, Kp optimization performed best overall (GMFE = 1.53) followed by logP optimization (GMFE = 1.69) and Rodgers equation (1.87). This is as expected, given the greater flexibility offered to optimization directly of the distribution-driving parameter Kp and removing the relativistic constraints between organs as a result of the Rodgers equation. Overall, Fig. 6 shows that median values were similar for all of the PK outputs, however the range of fold-errors was greater for Rodgers equation (non-optimized) especially for C_max_, where log(AFE) ranged from − 1.0 to 0.4. The interquartile ranges are centered around the median and log(Predicted/Observed) = 0, and there is a slight bias towards underprediction of all of the parameters as seen by the greater magnitude of negative log(AFE) compared to the maximum log(AFE). This is further confirmed with AFE values consistently less than 1; interestingly, direct Kp optimization showed a greater bias towards underprediction specifically of Vdss and MRT parameters (0.69, 0.61) compared to the other methodologies for prediction.

### Optimized distribution parameters

Figure 6 shows representative partition coefficients from the Rodgers equation for two organs with significantly different composition—muscle, gut—compared to their respective optimized (Kp) or hybrid-optimized (logP) values.

The regression fit is almost identical when comparing the two optimized distribution methods to the Rodgers calculation, however the actual tissue partition coefficients can vary dramatically. It is likely that the direct Kp-optimized distribution coefficient is in higher agreement with the logP-optimized one in the lipid rich organs as they drive systemic distribution. However, the agreement diminishes in other organ partition calculations (r^2^ = 0.95, 0.60 for gut and muscle, respectively). The absolute fold-difference between optimization techniques across the 21 compounds was 4.40-fold and 4.45-fold for muscle and gut, respectively, indicative of the fact that a global partition coefficient optimization can lead to accurate systemic but less accurate local outcomes. Overall, logP optimization was validated as a method for accurately optimizing compound distribution in silico while enabling organ-specific prediction of tissue partitioning.

## Discussion and conclusion

The BIOiSIM model successfully recapitulated systemic PK outputs using three different methodologies for optimizing distribution coefficients and through other parameters remaining mostly constrained by experimental datapoints. Several key considerations emerged from the analysis. The most readily apparent one is that different techniques for optimization of partition coefficients can present themselves as accurate on a systemic scale (plasma/blood) but vary significantly on a local scale (organ/tissue). As models for enabling better predictions of volume of distributions emerge^15, 16^, the translatability of these models between different species and specifically prediction of site-specific will need to be assessed through more robust methodologies, as mechanistic insights at an organ level may not be readily extractable. It is also critical to maintain an understanding of the original assumptions made in the mechanistic models when applying them in such a way. In the case of the current study, the Rodgers model is specifically applicable towards predicting steady-state partition coefficients in tissues based on their composition^22^. As non-lipid components have a significant contribution to the drug distribution in tissues a few more general models that use detailed organism and tissue compositions were proposed as for drug PK prediction as for simulation of the chemical toxicokinetic^43^. Tissues that have significant expression levels of influx/efflux transporters on their surface, such as the blood–brain barrier, are likely to require other models to expand beyond the assumed dominance of passive diffusion in compound permeation to the site^44^*.*

There are in vivo and in silico models used for the drug Kp predictions. The group of in vivo models includes Poulin and Theil model mentioned before that it uses linear regression to define the relationship between the in-vivo muscle Kpu value and the Kpu of all other tissues^21^, the Arundel multicompartment model that requires in-vivo VD_ss_ as an input parameter^45^, The Jansson model based on a combination of a measured volume of distribution and a lipophilicity descriptor of the compound that also uses linear regression analysis is used to predict Kp values for all other tissues from the in-vivo Kp value in muscle^46^. In-silico models are the Poulin et al. model incorporating two factors into the prediction of tissue:plasma partition coefficients—the solubility of a drug in lipids, and the binding of a drug to macromolecules^47^, the Berezhkovskiy model therefore represents a modified version of the Poulin et al. model, where tissue binding is being considered only in the water fraction^20^, and Rodgers-Rowland model predicting tissue:plasma water partition coefficients (Kp) for moderate-to-strong bases, and another to predict Kp for acids, very weak bases, neutrals and Group 2 zwitterions^22^. The latter model had been shown to be the most accurate a-priori model for the prediction of both Kp and Vss values. However, the model does show enough limitations to justify the further development and improvement of this method to increase its reliability and allow it to be used with more confidence during the drug development process^48^. Taking greater amounts of experimental parameter values such as chemical lipophilicity, p*K*a, phospholipid membrane binding, and the unbound plasma fraction, together with tissue fractions of water, neutral lipids, neutral and acidic phospholipids, proteins, and pH make Kp prediction more accurate across different species^49^. Although accuracy of those models is lower than accuracy of the direct Kp optimization using AI-based approach.

The framework that was utilized in this work was effective for testing the global translatability of mechanistic equations for distribution (specifically, Rodgers) and the method proposed can serve as a key driving force for testing and validating other mechanistic equations. It can address traditional issues with sensitivity of experimentally-measured parameters, and with conventions for translating physicochemical properties into physiologically relevant parameters (e.g. using vegetable oil partitioning for prediction of adipose tissue distribution instead of octanol–water partitioning)^50^. With the advent of ML/DL methods, further refinement can be undertaken on these types of optimized parameters. Future work can include studies that investigate sensitivity and optimization of other mechanistic equations for predicting distribution such as the derived Schmitt and Berezhkovsky models^20, 23^, and to explore the key assumptions around the driving forces of mechanisms such as compound ionization, hydrophilicity, and protein binding by optimizing p*K*a and plasma protein biding values instead.

ML algorithms that are well-suited for the prediction of the parameters are based on multiple factors, especially on the amount of the data and their characteristics. There has been good success in predicting physiological parameters using tree-based ensemble methods such as XGboost, random forest; support vector machines and Bayesian neural networks^51, 52^. These algorithms handle small datasets very well and avoid model overfitting. Different ML algorithms can be used for improving data robustness and reliability such as multivariate missing values imputation and feature engineering; model performance can be iterated through advanced algorithms on best model selection and hyperparameter optimization.

This work is a step towards driving the reliable prediction of distribution coefficients in tissues; a key requirement for reliable predictions of mechanistic PK behavior and disposition of xenobiotics^53^. This is particularly relevant to determination of first-in-human dosing regimens which are optimized to a target exposure level at a site-of-action based on available pharmacodynamic data. Thus, improved tissue distribution predictions can both improve translation of preclinical subject data and further refine human dosing based on distribution predictions beyond plasma PK^54^. ML optimization approaches such as the one presented here are a unique solution to the “missing data” problem, as they can be leveraged to fill-in the knowledge gaps using available data^53, 55^. For instance, in the present study proprietary ML algorithms were used to optimize Kp, k_a,_ and B:P values for testing compounds. This dataset can be rapidly expanded with other in vivo preclinical or clinical PK and by using parallelized simulation optimization, such as the one used by BIOiSIM. Additionally, population simulation studies can be applied for generating confidence intervals around the optimized mean parameters, further providing insight into the sensitivity of tissue-specific distribution to variance in tissue composition and general compound ADME behavior^56^. These same methodologies could also be used to generate confidence intervals for the simulation results through recursive sampling of parameter distributions to provide a more comprehensive assessment of simulation accuracy and variability.

The further expanded dataset of optimized values can be utilized to predict novel parameters with missing experimental data using QSPR/ML modeling. Unlike traditional QSPR modeling, ML-integrated models trained on both structural and mechanistic biological data would be theoretically expected to provide more accurate parameter predictions as the mechanistic descriptors can be expected to have a more definitive correlation with the parameter-of-interest^57, 58^. More comprehensive ML-based prediction pipelines that can select an optimal distribution model for specific structure-based clusters of compounds are also enabled through this type of validation approach.

## Methods

### Test dataset and data curation

For model building and validation, compounds were selected to ensure representation from basic, acidic, and zwitterionic chemical classes. The specific criteria for drug selection were:

1. Availability of in vivo plasma concentration–time profiles in healthy subjects for IV and/or Oral administration.
2. Experimental data for key PK parameters (protein binding, clearance, bioavailability for orally-administered compounds).
3. Experimental or computationally-generated values for logP (octanol–water partition coefficient) and p*K*a (disassociation constants).

A total of 21 structurally-diverse small molecule compounds that fulfilled the aforementioned criteria were chosen. Compound physicochemical properties and in vivo plasma concentration time profiles included in this study were extracted from publicly-available datasets published in peer-reviewed journals. The experimentally-determined parameters used as inputs for simulations are summarized in Supplementary Table S1.

Database for 21 test compounds contained 63 in vivo plasma concentration datasets for experimental drug parameters; the details of the datasets used are summarized in Supplementary Table S2. Traditionally, curating this dataset would be a time intensive task^29^. All of these variables can lead to the recording of factually-inaccurate information and significant knowledge gaps^16, 29^. Automated data processes present a more efficient, reliable, reproducible, and easily auditable method for data curation. Thus, the physicochemical, in vitro, and in vivo PK data obtained public sources were subjected to internal database consistency checks to evaluate data for robustness. These checks included: (1) comparison of calculated non-compartmental analysis metrics in the original publication to digitized values from the papers and (2) identification of experimentally-measured in vitro or in vivo parameters that violated physiological limitations (total clearance lower than cardiac output for the different subjects, Vd_ss_ greater than blood volume, B:P lower than 1 minus hematocrit).

### Subjects

Parameters characterizing subject-specific physiological behavior (e.g. organ blood flow rates, organ volume, tissue composition) for the different physiological compartments in the BIOiSIM model were adapted from reputed literature sources^30–32^. For the purpose of this study, compound datasets obtained in clinical studies were selected.

### The BIOiSIM model

**Figure 7:**
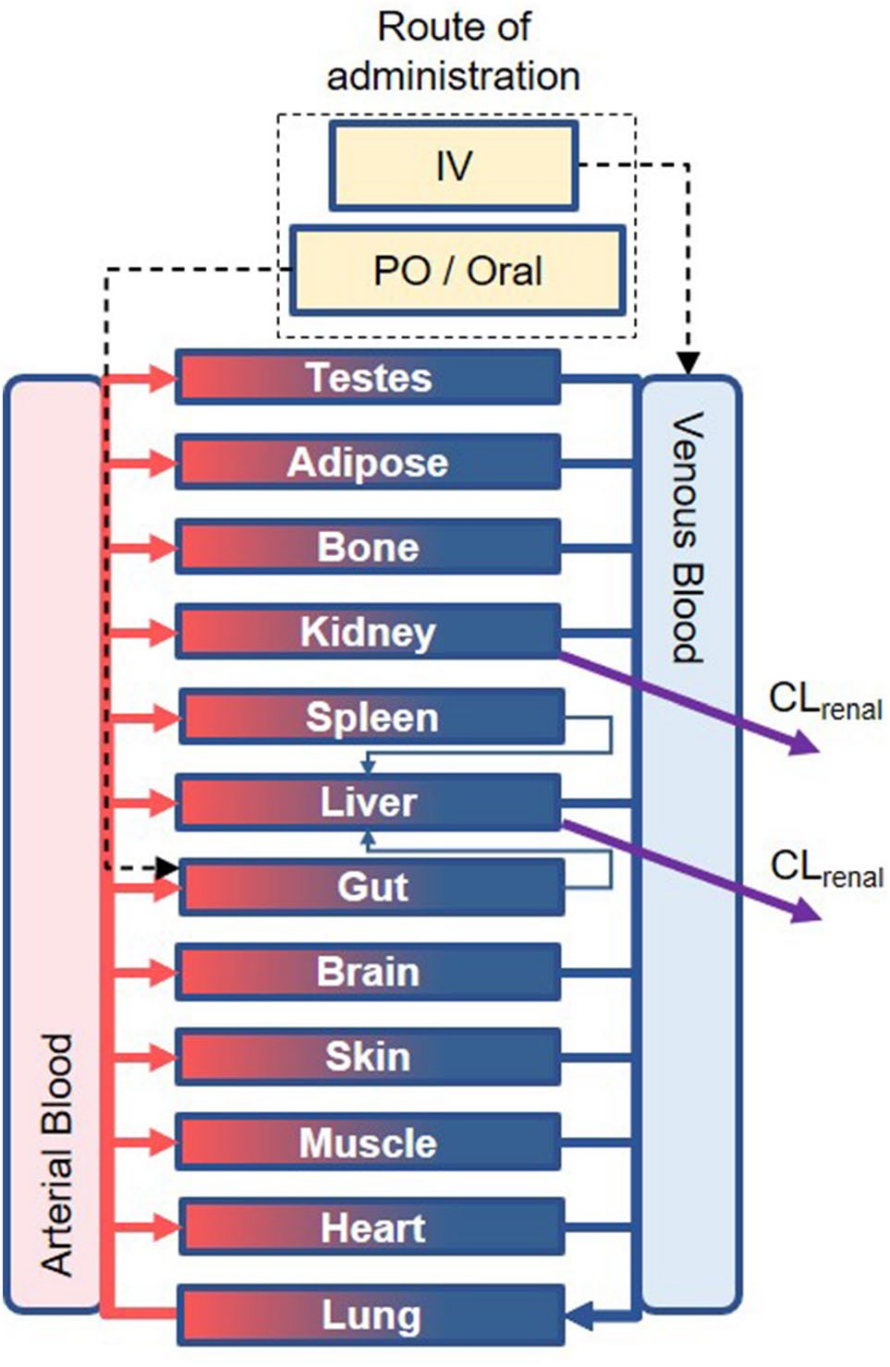
Overview diagram of the BIOiSIM mechanistic model. Note: some compartments are omitted for clarity. *CL*_*liver*_ hepatic clearance, *Cl*_*renal*_ renal clearance.

BIOiSIM is a patented AI biosimulation software platform, which includes a semi-mechanistic model of in vivo physiology with 14 individual compartments corresponding to important organs in the body (Fig. 7)^33^. The compartments are linked together using ordinary differential equations (ODE) as a function of tissue-dependent fluid dynamics, binding, partitioning and species-specific physiological characteristics^27, 28^. Model inputs include physiologically-specific parameters such as organ volumes, tissue composition, and blood flow rates, as well as mechanisms for clearance, drug solubility, and both intestinal and transdermal absorption. For this study, the implementation of the BIOiSIM model used was similar to commonly accepted physiologically-based pharmacokinetic (PBPK) models. Secondary inputs include drug-specific physicochemical properties and data such as in vitro microsomal clearances and fractions unbound in plasma. This model has been discussed in previous publications^27, 28^.

The mass balance between the compartments follows the general form:

1 $$V_{organ} \frac{{dC_{organ} }}{dt} = Q_{organ} \left( {C_{blood,in} - \frac{{C_{organ} }}{{K_{T:P,unbound} \times f_{unbound,plasma} }}\times B:P} \right)$$

For metabolizing and eliminating organs (e.g. liver, kidney) the general relationship is defined as:

2 $$V_{organ} \frac{{dC_{organ} }}{dt} = Q_{organ} \left( {C_{blood,in} - \frac{{C_{organ} }}{{K_{T:P,unbound} \times f_{unbound,plasma} }}\times B:P - CL_{organ} \times C_{organ} } \right)$$

where tissue-dependent parameters are expressed as *V* (organ volume), *Q* (flow rate), *CL* (organ-level clearance), and *K* (unbound tissue:plasma partition coefficient); *B:P* represents whole blood to plasma ratio, and *C* is the drug concentration in the specific compartment. The gut compartment includes a first-order model of intestinal absorption^34^. Hepatic metabolism and biliary clearance are modeled with a single clearance parameter, as neither in vivo sources of parameter values nor optimization sufficiently differentiate between the two routes of elimination.

The platform utilizes species-specific physiological parameters for the different organ compartments, as adapted from reputed literature sources^32, 35^. In addition to physiological parameters, the model inputs include drug-dependent PK (e.g. in vitro intrinsic clearance, B:P) and physicochemical (e.g. logP, p*K*a) parameters, and the in vivo study related information (dose, administration time, subject information, etc.) to simulate the experimental study as closely as possible. The drug-dependent parameters used in the model were either experimentally determined or optimized using a combination of ML cost minimization algorithms.

As is common with real-life data, experimental values for some drug parameters were unavailable. To fill-in these knowledge gaps, the ML framework is leveraged to optimize compound-specific PK parameters using existing in vivo study data. In the present work, this included tissue partitioning, first-order absorption rate constant (*ka*) and B:P, as reliable experimental measurements were unavailable^28^.

As stated previously, distribution is treated as an equilibrium partition coefficient of the form:

3 $$K_{Tissue:Plasma} = \frac{{C_{tissue} }}{{C_{plasma} }}$$

There are multiple different methodologies that have been utilized in an attempt to predict these partition coefficients accurately, including industry standards such as the Rodgers and Schmitt equations, however even these relationships are susceptible to significant variability^23, 36, 37^. These models predict compound partitioning as a function of logP (octanol–water partition coefficient), p*K*a, and plasma protein binding (f_up_); however, for specific groups of compounds they are found to underperform in their predictive capabilities based on the simplifying assumptions used^23, 36, 37^. We therefore compared the performance of the Rodgers model, optimization of the octanol–water partition coefficient as an input into the Rodgers model, and direct optimization of an average K_p_ to assess the accuracy of predicting in vivo human PK. The driving assumption was that mass balance mechanisms driving partitioning into tissues as described above are accurate, but the translation of physicochemical properties is not direct. Given that octanol–water partitioning is the highest sensitivity parameter for prediction of tissue-specific distribution, it was selected as the optimized parameter for testing of the Rodgers equation.

### Calculation of PK parameters

The in vivo plasma-concentration vs. time profiles were used to calculate the maximum plasma concentration (C_max_), area under the curve (AUC_0–t_, AUC_0–∞_), area under the moment curve (AUMC_0–t_). AUC and AUMC were calculated using the linear-trapezoidal method for experimentally-measured data^38^. Mean residence time (MRT) and volume of distribution at steady state (Vd_ss_) were calculated as:

4 $$MRT = \frac{{AUMC_{0 - \infty } }}{{AUC_{0 - \infty } }}$$

5 $$Vd_{ss} = \frac{Dose \times MRT}{{AUC_{0 - \infty } }}$$

The PK outputs from observed profiles and BIOiSIM-simulated plasma concentration profiles were calculated with the same methodology to increase confidence of comparison. Certain bounds were used as a reference to assess the physiological relevance of the PK outputs calculated from observed data. Specifically, for observed data, AUC_0–∞_ and dependent outputs were not calculated for cases where there were insufficient timepoints in the terminal elimination phase (plasma concentration < 0.25 × C_max_, or two half-lives; n = 11 compound simulation datasets excluded).

### Determination of objective functions

Adequate optimization of parameter values requires the utilization of an appropriate cost metric^39^. The behavior of a cost function should accurately reflect the convergence of optimization on discrete parameter combinations without overfitting to the explicit measurements^39^. The chosen accuracy measurement during this optimization was a function of the geometric means of C_max_, t_max_, and AUC_0-t_ and has been tested with an internal dataset of compound PK as it described in our previous work^27, 28^.

### Statistics and tools

The statistical methods utilized for assessment of model performance and optimization have been detailed in previous works^6, 28^. Briefly, in vivo plasma concentration datasets and associated error bars, when available, were manually digitized from source publications using "WebPlotDigitizer" version 4.2.34^40^. Model development and validation was done using the in-house platform in Python with Cython integration; matplotlib (v2.0.2) and Numpy (v1.14.2) were auxiliary packages used in simulation deployment and analysis. Model validation and analysis of model goodness-of-fit/accuracy was conducted using four quantitative metrics: absolute average fold error (AAFE), average fold-error (AFE), geometric mean fold-error (GMFE) across the pharmacokinetic outputs, and Pearson Correlation Coefficient (r^2^). The output parameters predicted specifically for this study using the BIOiSIM model include: C_max_, AUC_0–t_, AUC_0–∞_, AUMC_0–t_, MRT and Vd_ss_. Non-compartmental calculations were utilized to compare the accuracy of the simulations to the experimental data. For each PK output, AAFE and AFE were calculated as:

6 $$AFE = Average \;fold \;error = 10^{{\frac{1}{n}\mathop \sum \limits_{i = 1}^{n} \log \left( {\frac{{Predicted_{i} }}{{Observed_{i} }}} \right) }}$$

7 $$AAFE = Absolute \;average\; fold \;error = 10^{{\frac{1}{n}\mathop \sum \limits_{i = 1}^{n} \left| {\log \left( {\frac{{Predicted_{i} }}{{Observed_{i} }}} \right)} \right| }}$$

where *n* is the total number of compounds used in the analysis, and *Predicted*_i_/*Observed*_*i*_ correspond to predicted and observed values of PK parameters, respectively. The coefficient r^2^ was used to capture the overall trend in similarity between calculated and predicted parameters. Calculations of AFE, AAFE, GMFE, r^2^ and visual analysis were done in GraphPad Prism version 8.4.3 (GraphPad Software, San Diego, CA) and Microsoft Excel (2016).

Convergence plots were generated during optimization of the parameters to ensure adequate identification of absolute minima Afterwards, optimization algorithms were used to identify simulation parameters for logP, Kp, B:P, and k_a_^28, 41^. If the calculated and/or optimized values for tissue:plasma partition coefficients exceeded a maximum threshold (automatically calculated from experimental data), it was assumed that the calculation of the distribution coefficient was outside of physiologically-relevant range and an appropriate default value was assumed^42^. Each iterative simulation was deployed on the Amazon Elastic Compute Cloud (AWS EC2) and parallelized, allowing simultaneous output generation for all the compounds. Every hour of simulation (i.e. an hour of compound exposure in vivo) corresponded to approximately 0.08 s of computation time at a default step size of 0.0001 h (0.36 s).

## Supplementary Information

Supplementary Tables.
